# Toward the Use of Real‐World Data for Regulatory and HTA Decision‐Making: Experiences and Recommendations from Six EU‐Funded Projects

**DOI:** 10.1002/cpt.70502

**Published:** 2026-10-02

**Authors:** Martin Russek, Jonas Peltner, Kerstin Pfeifer, Theodoros N. Arvanitis, Dirk De Valck, Sieta T. de Vries, Michel Dumontier, Vera Ehrenstein, Omaima El Hakouni, Gregory Epiphaniou, Gokhan Ertaylan, Joana Fernandes, Scott Hansen, Stuart Harrison, Erzsébet Horváth‐Puhó, Saif Ul Islam, Pekka Kahri, Julia K. Mader, Carsten Maple, Nikolaos Matragkas, Shivani Misra, Peter G.M. Mol, Anne Paakinaho, Sami Pakarinen, Martina Radanović, Evy Reviers, Kathrine Kold Sørensen, Marco Spruit, Christina Tikka, Anna‐Maija Tolppanen, Christian Torp‐Pedersen, Mirna Vela, Thuan Vo, Puriya Daniel Würtz Yazdanfard, Britta Haenisch

**Affiliations:** ^1^ Research Division Federal Institute for Drugs and Medical Devices Bonn Germany; ^2^ Department of Electronic, Electrical and Systems Engineering, School of Engineering University of Birmingham Birmingham UK; ^3^ European Organisation for Professionals and People with ALS (EUpALS) Leuven Belgium; ^4^ Department of Clinical Pharmacy and Pharmacology University of Groningen, University Medical Center Groningen Groningen the Netherlands; ^5^ Department of Advanced Computing Sciences Maastricht University Maastricht the Netherlands; ^6^ Department of Clinical Epidemiology and Center for Population Medicine Aarhus University Aarhus N Denmark; ^7^ Division of Endocrinology and Diabetology, Department of Internal Medicine Medical University of Graz Graz Austria; ^8^ WMG University of Warwick Coventry UK; ^9^ Environmental Intelligence VITO (Flemish Institute for Technological Research) Mol Belgium; ^10^ INFARMED, National Authority of Medicines and Health Products, I.P, Information and Strategic Planning Department (DIPE) Lisbon Portugal; ^11^ The Open Group Berkshire UK; ^12^ ETHOS Digital Health Ltd Exeter UK; ^13^ HUS Helsinki University Hospital Helsinki Finland; ^14^ CEA, List Université Paris‐Saclay Palaiseau France; ^15^ Department of Metabolism, Digestion and Reproduction Imperial College London London UK; ^16^ School of Pharmacy, University of Eastern Finland Kuopio Eastern Finland Finland; ^17^ RISE ‐ Research and Innovation Services Göteborg Sweden; ^18^ Steno Diabetes Center Copenhagen Herlev Denmark; ^19^ Nordsjællands Hospital Hillerød Denmark; ^20^ Leiden University Medical Center Leiden South‐Holland the Netherlands; ^21^ Center for Translational Medicine, Medical Faculty University of Bonn Bonn Germany

## Abstract

Real‐world data are increasingly important for regulatory and health technology assessment decision‐making, yet their effective use across Europe remains limited by technical, methodological, regulatory, and societal challenges. Drawing on the experiences of six Horizon Europe projects in the MetReal cluster, this review summarizes common barriers and emerging solutions for the use of real‐world data in regulatory decision‐making. Key challenges include complex and lengthy data access procedures, fragmented governance frameworks, variable data quality and completeness, limited interoperability, methodological difficulties in analyzing heterogeneous datasets, and infrastructure constraints within secure data environments. The article highlights opportunities to address these barriers through improved metadata, harmonization, federated analytics, synthetic data sandboxes, sustainable funding, and stronger collaboration between researchers, regulators, health technology assessment bodies, data holders, and citizens. These recommendations aim to strengthen Europe's health data ecosystem and support more robust, efficient, and trusted evidence generation for public health and patient benefit.

Real‐world data (RWD) are increasingly recognized as a promising component of robust regulatory and health technology assessment (HTA) decision‐making, offering the potential to improve patient outcomes, and enhance public health. Europe is witnessing a surge in initiatives designed to unlock the value of RWD, including the DARWIN EU project^1^ and the European Health Data Space (EHDS)^2^. While these initiatives demonstrate significant ambition and offer benefits, challenges remain in for example, data quality, harmonization, interoperability, and governance^3^, ^4^. Most initiatives focus on technological solutions but the need for digital literacy among both healthcare professionals and patients is overlooked. Building trust with the public is also often neglected. There is a gap in ensuring that everyone can interact with and benefit from the health data landscape as well as room for more patient involvement in decision‐making^5^. On a technical level, different regulations, standards, and infrastructures for health data in different member states make it difficult to implement a cohesive framework for RWD use across the European Union (EU). Differences in data collection methods, data completeness, data governance and populations across countries can limit the comparability and reliability of the insights drawn^6^. Furthermore, there are gaps in the integration of new technologies such as artificial intelligence (AI) into the digital healthcare landscape, and infrastructure is still lagging behind hindering the use of AI in healthcare.^7^

To harness the potential of RWD, AI, and synthetic data for regulatory decision‐making and HTA, the EU recently provided funding to six projects within its Horizon Europe Programme (ID HORIZON‐HLTH‐2022‐TOOL‐11‐02): INSAFEDARE^7^, ^8^, ONCOVALUE^9^, ^10^, More‐EUROPA^11^, ^12^, Real4Reg^13^, ^14^, REALM^15^, ^16^, and REDDIE^17^, ^18^. These projects aim to facilitate the use of RWD in both regulatory approval decisions and HTA of medicinal products in Europe by identifying and addressing barriers in RWD use for regulatory decision‐making and HTA; by developing novel methods for the effective use of RWD; and by strengthening the patients' perspective in RWD research. The main aims of each project are shown in **Figure** **1**. To promote synergies, the projects established the MetReal (*Met*hods for analyzing *Real*‐world and synthetic data) cluster collaboration to share experiences and foster joint activities. Despite projects' diversity in coverage of areas of RWD applications in health care, some challenges are common across the projects. This paper aims to summarize these challenges and learnings by all projects and discuss them against the backdrop of current and future initiatives aiming to shape the European health data landscape. Based on the learnings, we provide recommendations for future research projects utilizing RWD within the EU. These recommendations address technical considerations, methodological challenges, and ethical and regulatory implications. Furthermore, targeted recommendations for funders and policymakers, advocating for strengthened investment in data findability and collection, data standardization and harmonization, and improved documentation will be presented.

**Figure 1:**
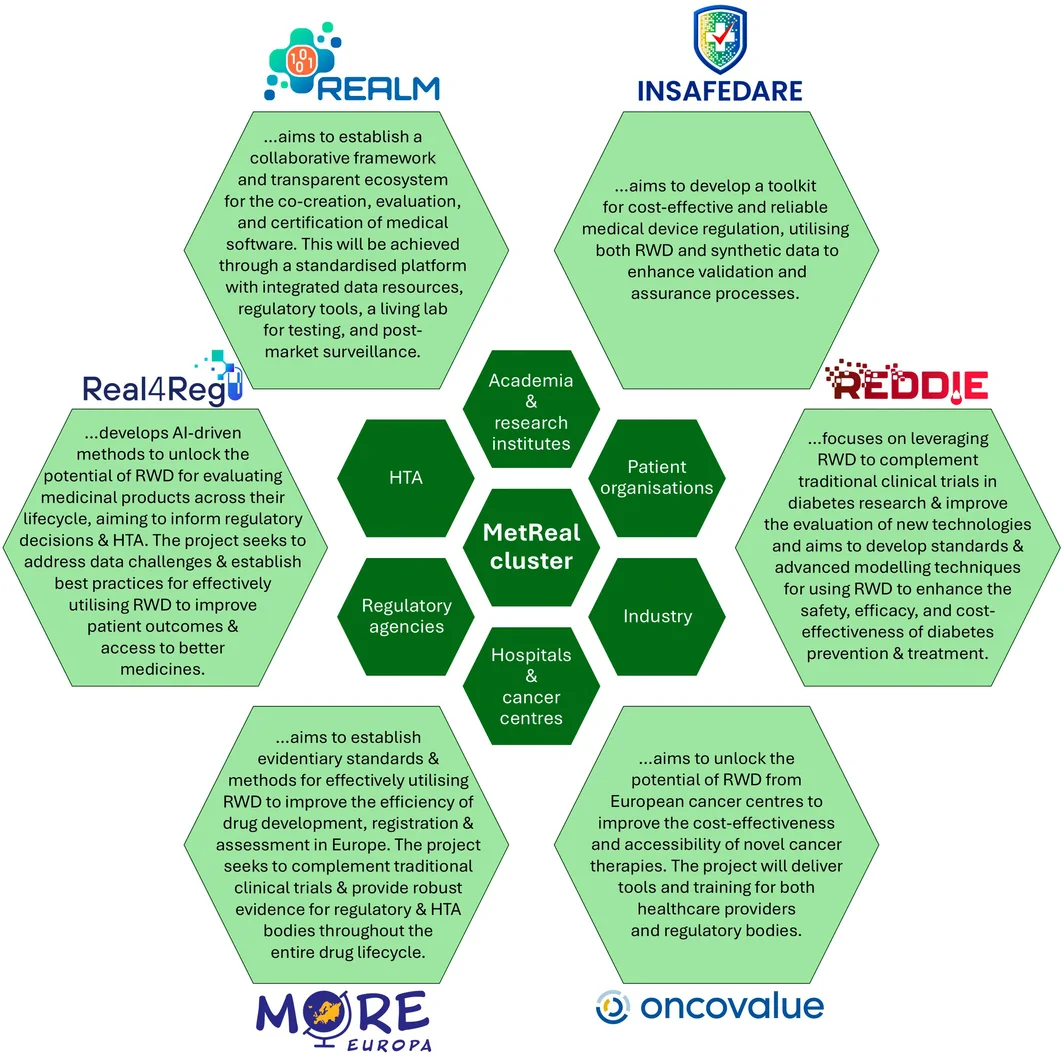
Main aims and stakeholder groups of the MetReal cluster projects. Dark green: Stakeholder groups involved in the MetReal cluster projects; light green: Main aims of each MetReal cluster project.

## EXPERIENCE: CHALLENGES AND LEARNINGS

During the projects, partners identified both challenges and opportunities relevant to RWD use and RWD synthetic data development. The major topics identified across the projects are data access, methodological challenges, and infrastructure, which are described in detail below. Some concrete examples of challenges and solutions are given in **Figure** **2**.

**Figure 2:**
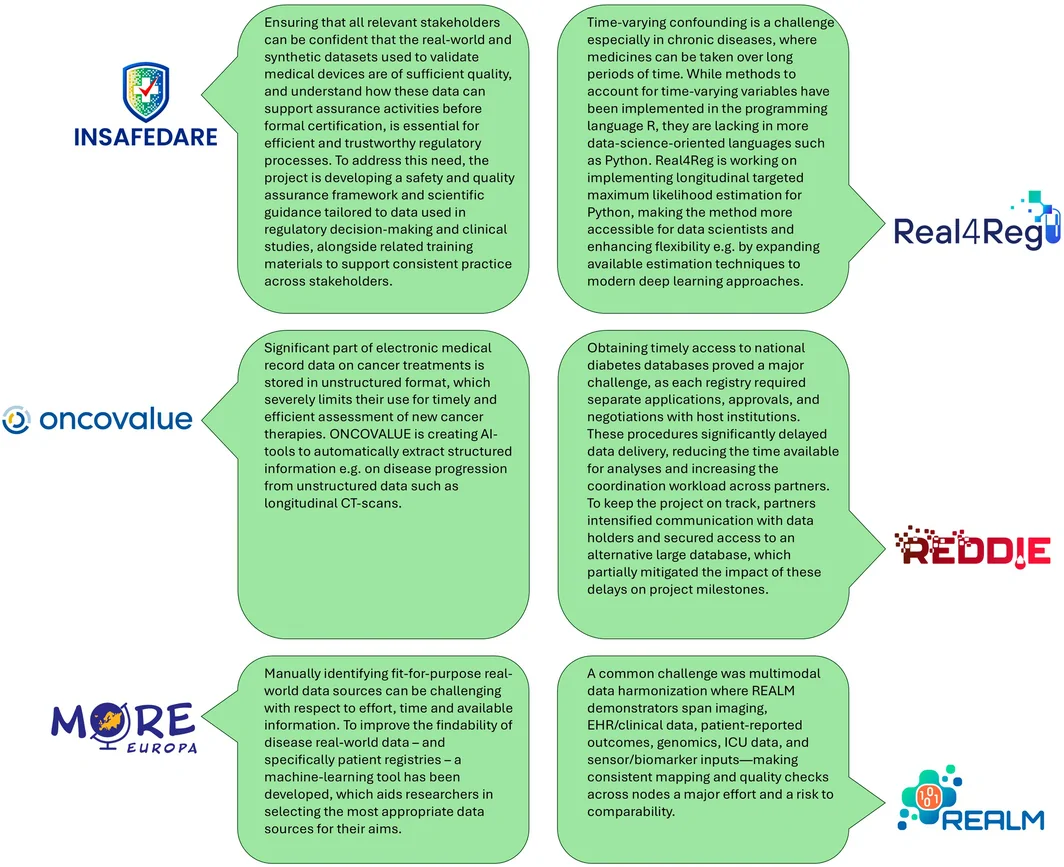
Examples of challenges and solutions provided by the MetReal cluster projects. CT: computed tomography. EHR: electronic health record. ICU: intensive care unit.

### Data access

One of the most encountered challenges was data access processes, which were often lengthy and complex. Administrative and legal procedures, including ethical approvals and data‐use agreements, often delay research timelines. Variability in national and institutional governance, different interpretations of EU data protection laws, and fragmented approval mechanisms add further complexity. In many cases, approval is required from multiple entities, and data‐use restrictions may prohibit export or cross‐border collaboration. Variations in data access models across countries and healthcare systems complicate multi‐database projects. In addition, costs and administrative fees associated with data access can be high, and long waiting times for data approval or linkage are common.

### Methodological challenges

RWD enable powerful research, but they also pose several methodological challenges. Their primary strength, which is based on the prospective collection of clinical or administrative data and hypothesis‐free data accrual, can lead to bias. Researchers often face inconsistent levels of transparency in data governance and limited insight into how data are collected, curated, updated or validated, which affects data comparability and data quality. Missing data are common, and unclear data provenance can make statistical correction methods such as imputation challenging. Provenance also influences key data quality parameters, including completeness, sensitivity, and positive predictive value. Semantic barriers such as differences in variable definitions and coding systems, as well as data completeness across data sources increase the workload for harmonization and can limit cross‐study comparability. Changes in coding practices (e.g., diagnostic classifications) over time further complicate longitudinal analyses. In some datasets, biologically or clinically important variables are not captured because data was collected for local administrative rather than research purposes. Coverage can also change over time.

Researchers also face complex analytical challenges such as handling time‐varying exposures, competing risks and immortal time bias. While registry studies provide strong real‐world evidence (RWE), causal inference can be limited without robust study designs, appropriate sensitivity analyses and validation. Data linkage on the level of individuals is not always possible, constraining the ability to integrate information from various domains (e.g., pharmacy, clinical, and outcomes data). Moreover, the heterogeneity of data types in medical settings makes it highly challenging to design methods capable of accurately extracting meaningful information from multimodal inputs.

### Infrastructure

Infrastructure was identified as a critical factor in both enabling and limiting effective use of RWD. On the positive side, many systems maintain secure, high‐quality databases that allow longitudinal tracking of patients and outcomes. The structured collection and maintenance of these data contribute to validity and reliability, supporting benchmarking, quality improvement, and research collaborations. However, major technical and logistical challenges persist. Secure data processing environments enhance privacy but can create bottlenecks as every software installation, data extraction, synchronization, or output export must be approved, which can cause delays. Computing environments often lack flexibility, with limited availability of programming languages, software libraries, or data analysis tools. Heterogeneous infrastructures across data sources add to the complexity of multi‐site analyses. Processing very large datasets requires high computing power and storage space, which are not always available in the environments where data are hosted. Restrictions on remote access or limitations on who can analyze the data further reduce efficiency. When analyses require multiple researchers or parallel computation, these data access restrictions can severely slow progress.

## OUTLOOK: RECOMMENDATIONS FOR THE FUTURE

The experiences collected so far within the six MetReal projects have led to the formulation of recommendations for the use and analysis of RWD for several stakeholders in the health care sector. Together with some of the solutions provided by MetReal cluster projects (See **Figure** **2**), these can help to advance RWD research across Europe and improve the evidence base for regulatory decisions and HTAs.

### EU policy level

Enhanced support for data holders is needed to improve documentation, data quality, and AI readiness of data sources. Specifically, this might include (1) one‐off grants for the acquisition of state‐of‐the‐art computing infrastructure^19^ to facilitate federated data analysis and AI‐based research, (2) continuous funding to improve coverage of data sources that are crucial for public health research, or (3) EU‐level projects for the standardization and translation of metadata^20^.

Another important area is the active collection of knowledge about data sources within the emerging EHDS. Every EHDS‐aligned research project, regardless of its outcome, is generating a plethora of information about data sources' coverage, unexpected sources of bias, or might be able to clinically validate certain outcomes. While some of that information will be published in project reports and scientific literature, much of it will be lost over time. Establishing a process for collecting and exchanging this information—whether as part of the metadata of individual data sources, centrally via the HMA‐EMA RWD catalogue^21^, or additionally via multistakeholder platforms—will bring multiple benefits: It will improve the validity of scientific research, help regulatory and HTA bodies to better evaluate the quality of evidence, and increase public trust in results from well‐documented data sources.

In parallel with the implementation of the EHDS for secondary use through health data access bodies (HDABs), the possibilities of federated data processing and analysis should be pursued whenever possible. Although federated data models may not yet meet the most rigorous analytical and evidentiary standards, the evolving possibilities of federated analytics, as well as AI tool validation, across different geographical settings will allow data exploitation without person‐level data transfer. The idea of federated data models alongside data storage and control by healthcare institutions should play an important role in the European health data landscape, as has been highlighted by early flagship projects^22^.

### Health data access bodies and data holders

HDABs and data holders need to collaborate to provide researchers with transparent information on data structure and content. This will significantly reduce the burden on many stakeholders by facilitating feasibility checks and the selection of data sources: If feasibility checks no longer need direct data access, HDABs will have fewer applications to process for such studies, and data holders require fewer resources to conduct the applicants' feasibility studies. Researchers will be able to better plan their studies and thus save time and resources, and the public will have more trust in results as more information on the data sources is publicly available.

Similarly, data holders who offer a sandbox^23^ with realistic synthetic data would not only reduce the computational burden on the real‐data analysis platforms but also accelerate the analysis process for data users. Researchers could prepare analysis scripts before accessing data and would only need to make minimal adjustments later, reducing the time spent running incomplete scripts.

Lastly, there are several ways to improve efficiency in data access applications:

HDABs should be encouraged to provide best practice examples of successful applications, highlighting what level of detail needs to be provided for an application to be processed smoothly. These should be kept up to date as application procedures change. Similar recommendations apply to patient registries that generally have a more curated and structured data collection.

Application forms should be aligned with common study protocol templates such as the HARmonized Protocol Template to Enhance Reproducibility of Hypothesis Evaluating Real‐World Evidence Studies on Treatment Effects (HARPER)^24^, to reduce the burden on applicants. The possibility of a parallel ethics approval process should be considered.

Since timelines for regulatory/HTA decisions are often relatively short^25^, studies on specific procedures must be approved quickly to ensure that regulatory authorities and HTA bodies can use the EHDS and incorporate current RWE into their decisions.

To reduce the burden on individual HDABs and fully embrace the EHDS, the implementation of “centralised application procedures” should be considered for multi‐country studies. The HealthData@EU central platform^26^ could have the potential as a facilitator for this procedure.

### Funders of RWD projects

To improve RWD research quality in Europe, funders are also encouraged to adapt their funding strategies. As could be seen across all MetReal projects, data access applications currently take too long in relation to the project duration. Flexible handling of project start dates would make it possible to coordinate the start of research activities with data access in future projects. Funders should also allocate more resources for sustainability—this includes coordination with other projects and initiatives and long‐term support to keep code/tools and information up to date after the official end of a project.

When developing new analytical methods, one of the biggest challenges is establishing these methods in applied research in competition with commonly used but less suitable methods. One example is the analysis of adverse events as raw proportions rather than time‐to‐event data^27^. To improve the sustainability of project resources and enhance overall research quality in the future, particular attention should therefore be paid to cooperation initiatives that aim to apply newly developed validated methods and establish them as the standard approach for analysis, replacing outdated, suboptimal methods. Particularly, collaboration with regulatory and HTA bodies can help to ensure the best methods are included in guidance documents, as was the case with target trial emulation in the National Institute for Health and Care Excellence (NICE)'s RWE framework^28^.

### RWD community

For the community of RWD researchers, there are several topics that warrant further attention to advance the field. In line with the need for improved computational infrastructure, developers of data analytics methods should also aim to improve the computational efficiency of these methods. Since RWD often have substantial size, any reduction in computing time^29^ and/or usage of memory during computation^30^ can make application of advanced analytical tools more feasible. To enable standardized assessment of data quality, emphasis should also be placed on defining concrete data quality metrics to be achieved by data holders. Similarly, establishing quality control procedures for novel methods, including AI techniques, would help the acceptance of those methods across stakeholder groups.

Research on heterogeneity of results from multi‐database RWD studies is still far too limited. This research requires cooperative efforts between many research institutions, and the use of findings from many past, ongoing, and future RWD research projects. Understanding the causes of heterogeneity from different RWD sources and the ability to predict them will be crucial to increasing regulatory acceptance of RWD in the future by either being able to reduce or make use of this heterogeneity.

Finally, informing and involving citizens is crucial. Generalizability is one of the key features of RWD, and this can only be achieved if the public allows their data to be used for research purposes. Although patients are generally willing to share their data, educating and informing the public about the purposes, data protection, and implications of RWD research will play an important role in maintaining public trust.

These recommendations aim to strengthen the foundational conditions necessary to support robust evidence generation for future regulatory and HTA decision‐making. Alongside efforts to improve the upstream conditions for evidence generation, as detailed in this article, differences in the interpretation of real‐world evidence (RWE) between regulatory and HTA agencies represent an additional source of complexity^31^ emphasizing the need for multistakeholder dialog.

## CONCLUSION

Although many challenges related to RWD‐based research can be identified, solutions to most of them are available and within reach. It is necessary to support and encourage initiatives to improve the current situation, whether through funding, legislation, collaboration, or research. By working together toward this goal, the European community can have a lasting impact on the efficient and responsible use of citizens' health data, thereby improving the evidence base for regulatory decisions and HTAs, and thus the availability of safe and effective treatments for patients not only in Europe but also worldwide.

## Funding

The research leading to this manuscript has been funded through Horizon Europe grant agreement numbers 101095353 (Real4Reg), A101095556 (REDDIE), 101095435 (REALM), 101095479 (More‐EUROPA), 101095245 (ONCOVALUE), and 101095661 (INSAFEDARE). The funder did not play a role in study design, execution, or interpretation.

## CONFLICTS OF INTEREST

JKM is a member of advisory boards of Abbott Diabetes Care, Becton‐Dickinson, Biomea Fusion, Dexcom, Eli Lilly, Embecta, Insulet, Medtronic, Novo Nordisk A/S, Pharmasens, Roche Diabetes Care, Sanofi‐Aventis, and Tandem. She has received speaker honoraria from A. Menarini Diagnostics, Abbott Diabetes Care, Buzud, Dexcom, Embecta, Eli Lilly, Novo Nordisk A/S, Roche Diabetes Care, Sanofi, Sinocare, Viatris, and Ypsomed. JKM is also a shareholder of decide Clinical Software GmbH and elyte Diagnostics and serves as CMO of elyte Diagnostics. CTP reports grants for studies from Bayer and Novo Nordisk. SM has received honoraria for educational talks from Sanofi, Lilly, and Menarini, and Advisory Boards from Insulet. All other authors reported no conflict of interest.
